# Comparison of prognostic outcomes between endoscopic submucosal dissection and surgical treatment for early gastric cancer: a retrospective cohort study

**DOI:** 10.1186/s12876-024-03186-y

**Published:** 2024-03-04

**Authors:** Yifan Zhang, Fangzhen Shi, Yuxiang Fan, Gang Liu, Chengkai Xia, Haodong Wang

**Affiliations:** 1 Department of Gastrointestinal Surgery, Affiliated Kunshan Hospital to Jiangsu University, 215300 Suzhou, Jiangsu China; 2 Department of Gastroenterology, Kunshan Sixth People’s Hospital, 215321 Suzhou, Jiangsu China; 3 Department of General Surgery, Kunshan Sixth People’s Hospital, 215321 Suzhou, Jiangsu China; 4 Department of Emergency Surgery, Affiliated Kunshan Hospital to Jiangsu University, 215300 Suzhou, Jiangsu China

**Keywords:** Early gastric Cancer, Endoscopic submucosal dissection, Prognosis, Surgery

## Abstract

**Background and aim:**

The optimal management strategy for early gastric cancer (EGC) a topic of contention. This study aims to compare the prognostic outcomes of endoscopic submucosal dissection (ESD) and surgical treatment in patients diagnosed with EGC.

**Methods:**

In thisretrospective cohort study, we analyzed data from539 patients diagnosed with EGC between January 2012 and December 2020 from two centers. We compared Clinicopathological features, procedure-related complications, recurrence rate, overall survival, and disease specific survival between the 262 patients who underwent ESD and the 277 patients who underwent surgical treatment. ESD procedures were conducted using a dual knife by experienced endoscopists, while surgical treatments included laparoscopic or open gastrectomy. Regular ollow-up examinations were conducted post-treatment.

**Results:**

The two groups exhibited comparable baseline characteristics. Multivariable Cox regression analysis identified vascular invasion as a risk factor for worse recurrence-free survival (RFS), and overall survival (OS) in patients with early gastric cancer. The ESD group experienced fewer overall postoperative complications compared to the surgical treatment group. Kaplan-Meier curves demonstrated no significant differences in recurrence rate or overall survival between the two groups.

**Conclusions:**

Both ESD and surgical treatment emerged as safe and effective approaches for managing EGC. The choice of treatment should be tailored to individual patient factors. ESD can be considered an alternative treatment option for selected patients who are not suitable candidates for surgery. Further studies are warranted to determine the long-term outcomes of ESD and surgical treatment for EGC.

## Introduction

Early gastric cancer (EGC) refers to a type of gastric neoplasm that is limited to either the mucosa or submucosa layer, and is not accompanied by lymph node metastasis [1, 2]. EGC has a much better prognosis compared to advanced gastric cancer (AGC), with a 5-year survival rate of more than 90% after curative resection. Endoscopic submucosal dissection (ESD) has been increasingly used as a minimally invasive treatment for EGC, as it allows for en bloc resection of larger and more complex lesions compared to conventional endoscopic mucosal resection (EMR) [3, 4]. However, there is still ongoing debate regarding the long-term clinical outcomes of ESD compared to surgical resection.

While ESD is associated with lower morbidity rates and shorter hospital stays compared to surgical resection, concerns remain about its potential for incomplete resection and higher recurrence rates. Therefore, it is important to determine whether ESD can achieve equivalent long-term outcomes in terms of recurrence and survival rates compared to surgical resection [5, 6].

Several studies have investigated the efficacy and safety of ESD compared to surgical resection in the treatment of EGC. However, most of these studies have focused on short-term outcomes, and there is still limited data on the long-term outcomes of ESD compared to surgical resection, particularly in terms of recurrence rates and overall survival [7, 8].

Therefore, the aim of this study is to compare the long-term clinical outcomes of ESD and surgical resection in patients with EGC, with a specific focus on recurrence rates and overall survival. Our hypothesis is that ESD will have comparable long-term outcomes to surgical resection in the treatment of EGC, validating its use as a less invasive alternative to surgery. By comparing the two techniques, we hope to provide clinicians with valuable information to guide treatment decisions for patients with EGC.

## Materials and methods

### Patient selection

We enrolled a total of 539 patients with early gastric cancer (EGC) who underwent endoscopic submucosal dissection (ESD) or surgical treatment from two medical centers - Affiliated Kunshan Hospital to Jiangsu University and Kunshan Sixth People’s Hospital, between January 2012 and December 2020. The inclusion criteria applied were strictly followed for all patients from both centers: (1) confirmed diagnosis of EGC; (2) postoperative pathology reports jointly determined as gastric cancer by two experienced pathologists; (3) preoperative American Society of Anesthesiologists (ASA) score ≤ II; (4) Eastern Tumor Collaborative Group score ≤ 2; (5) first tumor findings; (6) no prior treatment. Exclusion criteria were as follows: (1) advanced gastric cancer; (2) prior treatment other than ESD or surgery; (3) incomplete clinicopathological data; (4) non-curative resection This retrospective study was approved by the Ethics Committees of Affiliated Kunshan Hospital to Jiangsu University and Kunshan Sixth People’s Hospital and adhered to the ethical principles outlined in the Declaration of Helsinki. Informed consent forms were obtained from all patients.

### Pathological investigation methods

#### Specimen collection

##### Endoscopic submucosal dissection (ESD)

Specimens were obtained through ESD procedures performed by experienced endoscopists.

A dual knife was used for ESD.

##### Surgical treatment

Specimens were collected during laparoscopic or open gastrectomy procedures.

#### Pathological examination

##### Histopathological evaluation

All specimens underwent thorough histopathological examination.

Evaluation of tumor size, depth of invasion, lymphovascular invasion, and other relevant features.

#### Lymphatic invasion assessment

##### Inclusion in histopathological evaluation

The evaluation of lymphatic invasion was an integral part of the histopathological examination.

Specific staining techniques (e.g., immunohistochemistry) were employed to identify lymphatic invasion, ensuring comprehensive assessment.

#### Depth of submucosal (SM) invasion

1. Methods for Depth Determination.

##### Imaging modalities

Preoperative imaging modalities, including endoscopic ultrasound (EUS) and computed tomography (CT), were utilized to assess the depth of invasion.

(2) Intraoperative Assessment:

During ESD and surgical procedures, real-time intraoperative assessments were made to confirm and further detail the depth of invasion. During ESD procedures, real-time intraoperative assessment involves continuous monitoring of the cutting process to determine the depth of tumor invasion. Endoscopists first carefully examine the lesion using high-definition endoscopy to observe its invasion depth within the gastric mucosa or submucosa. They then utilize magnifying endoscopy with narrow-band imaging (NBI) to enhance visual acuity and accurately delineate the lesion’s borders. Additionally, the use of absorptive dyes such as indigo carmine or crystal violet during chromoendoscopy aids in outlining tumor margins and identifying any tiny infiltration areas beyond the mucosal layer. Intraoperative assessment during surgery includes tactile feedback from the operating surgeon and visual inspection. Palpation of the gastric wall can reveal thickening or nodules at any lesion site, indicating the possibility of submucosal infiltration. Endoscopic examination can also be performed during surgery to confirm the tumor’s location and extent, especially in cases where preoperative endoscopic findings are inconclusive.

The staging criteria adopted was developed by the Japanese Gastric Cancer Association (JGCA). Early gastric cancer is classified based on the depth of infiltration into mucosal or submucosal layers, denoted as M and SM, respectively. Mucosal cancer is further divided into M1, M2, and M3. M1 refers to intraepithelial cancer, limited to the superficial layer of the submucosa. M2 indicates slightly deeper infiltration, reaching the middle layer of the submucosa. M3 involves further penetration into the deep layer of the submucosa. Submucosal cancer is categorized as SM1, SM2, and SM3 based on increasing depth of infiltration. SM1 denotes cancer cells infiltrating the upper third of the submucosal layer, SM2 involves infiltration into the middle third, and SM3 signifies infiltration into the lower third of the submucosal layer.

### Pre-operation preparation and operation procedure

All physicians crossed the learning curves for both surgery and ESD. Prior to ESD or surgical treatment, all patients underwent a comprehensive evaluation to ensure that they met the preoperative criteria for their respective procedures. This included a thorough assessment of medical history and physical examination, as well as preoperative laboratory testing such as complete blood count, liver and kidney function tests, and coagulation function tests. Additionally, preoperative imaging examinations were conducted, including abdominal ultrasound, enhanced abdominal CT, and abdominal MRI to assess the resectability of the tumor.

For ESD, the indications are mainly intramucosal (M1) and superficially infiltrated submucosal (SM1).Before the procedure, a detailed endoscopic evaluation is performed prior to the procedure to determine the characteristics of the lesion, including location, size, depth of invasion, and macroscopic type. The treatment approach is then determined based on these factors, with consideration given to the patient’s overall health status. During the ESD procedure, which is performed under sedation or general anesthesia with the patient in a supine position, an endoscope is inserted through the mouth to reach the stomach. A specialized device called a dual knife is then used to perform precise dissection of the lesion from the surrounding tissue. The mucosa and submucosa are dissected en bloc, and hemostasis is achieved using electrocoagulation to control bleeding.

For EGC, the decision to perform laparoscopic or open gastrectomy is made after a comprehensive consideration of factors such as tumor size, location, and depth of invasion, as well as the patient’s overall health status. Hemostasis is maintained throughout the procedure to control bleeding and ensure optimal outcomes.

Patients are placed under general anesthesia, and a midline incision or several small incisions are made in the abdomen. The surgeon will then proceed to remove part or all of the stomach, depending on the location and size of the tumor, as well as other factors such as the presence of lymph node metastasis. Reconstruction of the gastrointestinal tract is performed using various techniques, including gastroduodenostomy, gastrojejunostomy, Roux-en-Y esophagojejunostomy, or Billroth I or II reconstruction, depending on the extent of resection required.

### Variables

Based on the patients’ basic characteristics as well as tumor characteristics and immunohistochemical features, we collected 13 variables, including gender, age, American Society of Anesthesiology (ASA) score, ECOG PS, Family history of gastric cancer, Tumor location, Tumor size, Differentiation, Vascular invasion, P53, Ki-67, Tumor infiltration, and MSI status.

### Definition

After surgical treatment or endoscopic submucosal dissection (ESD) for early gastric cancer (EGC), patients were followed up by two professional followers at our medical center. Follow-up visits were conducted every three months in the first year after discharge, and every six months from the second year.

During each follow-up visit, enhanced CT and abdominal MRI scans were performed, with PET-CT scans conducted for necessary patients. Laboratory tests such as liver function, kidney function, and tumor markers were also conducted to monitor the patient’s overall health status.

Overall survival (OS) was defined as the time between the first postoperative day and the date of death, while recurrence-free survival (RFS) was defined as the time between the first postoperative day and the date of recurrence (defined as new organisms detected on postoperative imaging). The follow-up period ended on January 30, 2023.

### Data analysis

All dichotomous variables were subjected to statistical analysis using the chi-square test or Fisher’s exact test. Kaplan-Meier survival curves were plotted, and statistical tests were performed using the log-rank method. Univariate Cox regression analysis was performed on variables with *p* < 0.05, which were then included in multivariate regression analysis to identify prognostic factors.

Statistical aanalysis was conducted using SPSS version 25.0 (IBM, Armonk, New York, USA), and a two-tailed p-value < 0.05 was considered statistically significant. R software version 4.0.5 (R Statistical Computing Project, Vienna, Austria) was used to plot all survival curves.

Sample size estimation was conducted using PASS version 11.0 prior to the commencement of the study. Additional details regarding the sample size calculation, including power analysis and effect size, are available upon request.

## Results

### Inclusion of exclusion process

**Fig. 1:**
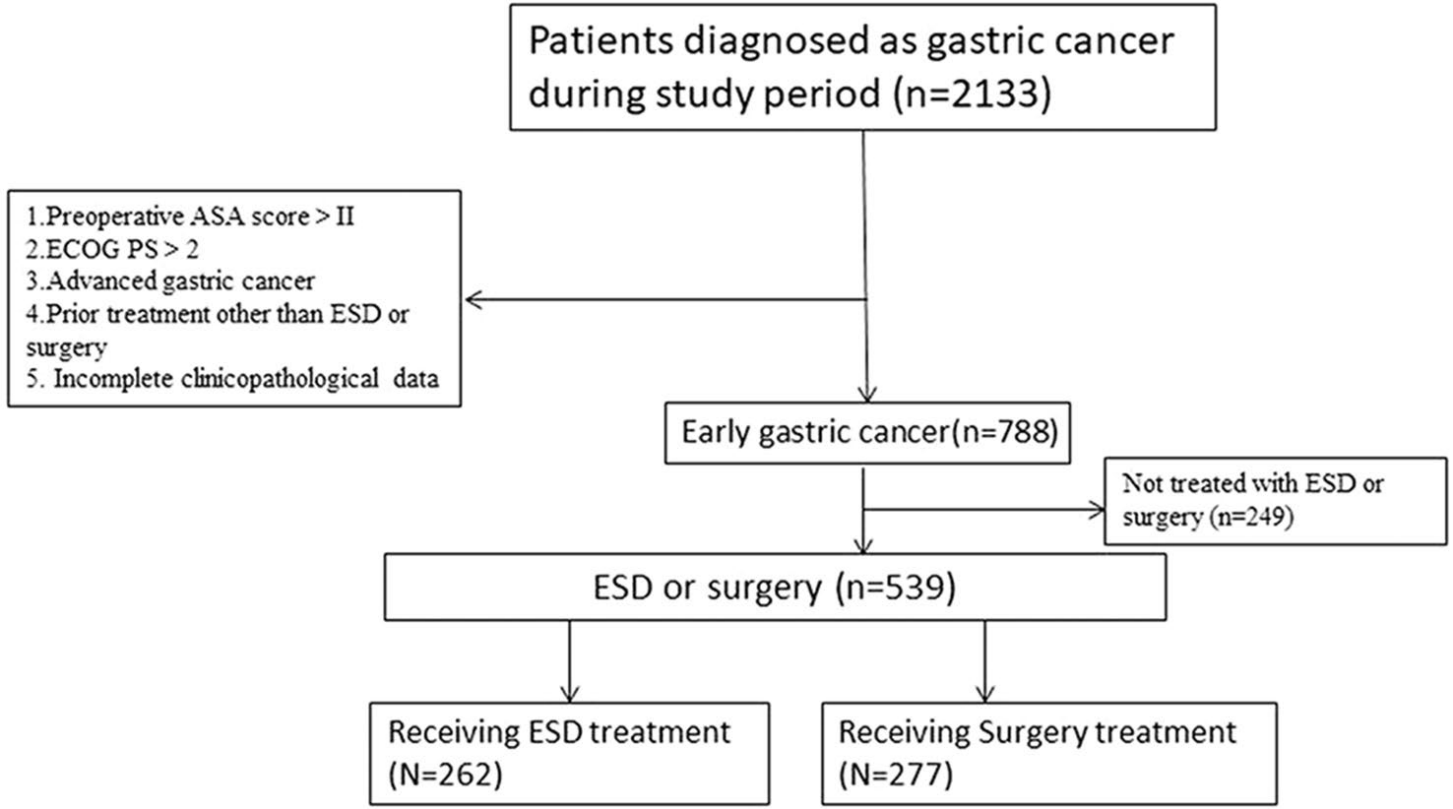
Flow chart for inclusion and exclusion of patients with early gastric cancer who underwent ESD or surgery

A total of 2133 patients initially met the basic requirements during the study time period, and a total of 539 patients were finally identified to receive the ESD and Surgery groups based on strict inclusion and exclusion criteria, as detailed in the process in Fig. 1.

### Baseline information of patients in the training and validation groups

Baseline characteristics of patients in the ESD group (*n* = 262) and surgical group (*n* = 277) were compared using statistical tests. No significant differences were observed in the gender distribution, with 77.9% of patients in the ESD group being male compared to 84.5% in the surgery group (*p* = 0.060). Similarly, there was no significant difference in age distribution, with 57.6% of patients in the ESD group being under 60 years old compared to 55.6% in the surgery group (*P* = 0.664).

There were also no significant differences in ASA physical status classification, with 89.7% of patients in the ESD group and 87.4% in the surgery group classified as ASA grade I (*p* = 0.420). The majority of patients in both groups did not have a family history of gastric cancer, with 87.0% of patients in the ESD group and 90.6% in the surgery group reporting no family history (*P* = 0.151).

No significant differences were observed between the two groups in terms of tumor location or size, with 37.0% of tumors in the ESD group located in the body compared to 36.8% in the surgery group (*P* = 0.812), and 58.0% of tumors in the ESD group measuring ≥ 2 cm compared to 53.4% in the surgery group (*P* = 0.299). Moreover, there were no significant differences in tumor differentiation, vascular invasion, tumor infiltration, or MSI status. Similarly, in the past medical history, such as cardiovascular diseases, liver diseases, kidney diseases, respiratory system diseases, and diabetes, there were no statistically significant differences between the two groups **(**Table 1**)**.

**Table 1:** Baseline characteristics of patients with gastric cancer undergoing ESD or gastrectomy (*n* = 539)

		ESD(*n* = 262)	Surgery(*n* = 277)	P-value
Gender(%)				0.060
	Male	204(77.9)	234(84.5)	
	Female	58(22.1)	43(15.5)	
Age(%)				0.664
	< 60 y	151(57.6)	154(55.6)	
	≥ 60 y	111(42.4)	123(44.4)	
ASA(%)				0.420
	I	235(89.7)	242(87.4)	
	II	27(10.3)	35(12.6)	
ECOG PS(%)				
	0	210(80.2)	221(79.8)	
	1	52(19.8)	56(20.2)	
Family history of gastric cancer(%)				0.151
	Yes	34(13.0)	26(9.4)	
	No	228(87.0)	251(90.6)	
Tumor location(%)				0.812
	Cardia	53(20.2)	58(21.0)	
	Fundus	62(23.7)	67(24.2)	
	Body	97(37.0)	102(36.8)	
	Antrum	50(19.1)	50(18.0)	
Tumor size(%)				0.299
	< 2 cm	110(42.0)	129(46.6)	
	≥ 2 cm	152(58.0)	148(53.4)	
Differentiation(%)				0.678
	High	77(29.4)	86(31.1)	
	Moderate	139(53.0)	142(51.3)	
	Poor	46(17.6)	49(17.7)	
Vascular invasion(%)				0.715
	No	178(67.9)	184(66.4)	
	Yes	84(32.1)	93(33.6)	
P53(%)				< 0.001
	No	155(59.2)	121(43.7)	
	Yes	107(40.8)	156(56.3)	
Ki-67(%)				0.814
	< 50%	162(61.8)	186(67.2)	
	≥ 50%	100(38.2)	91(32.8)	
Tumor infiltration(%)				0.874
	Mucosa	97(37.0)	96(34.7)	
	Submucosa SM1	165(63.0)	181(65.3)	
MSI status(%)				0.139
	MSS/MSI-low	237(90.5)	257(92.8)	
	MSI-high	25(9.5)	20(7.2)	
Cardiovascular disease				0.532
	No	170(64.9)	172(62.1)	
	Yes	92(35.1)	105(37.9)	
Liver disease				0.369
	No	249(95.0)	258(93.1)	
	Yes	13(5.0)	19(6.9)	
Renal disease				0.146
	No	254(96.9)	261(94.2)	
	Yes	8(3.1)	16(5.8)	
Respiratory disease				0.568
	No	255(97.3)	272(98.2)	
	Yes	7(2.7)	5(1.8)	
Diabetes				0.465
	No	227(86.6)	233(84.1)	
	Yes	35(13.4)	44(15.9)	

ESD: Endoscopic Submucosal Dissection; ASA: American Society of Anesthesiologists; ECOG: Eastern Cooperative Oncology Group; PS: performance status; MSI: microsatellite instability; MSS: microsatellite stable

TNM stages are according to AJCC 8th edition

### Univariate and multivariate Cox regression analysis of predictors for gastric cancer patient survival time

In the univariate analysis, we found that tumor size greater than 2 cm (HR = 1.569, 95% CI = 1.062–2.318, *P* = 0.024), poorly differentiated tumor (HR = 1.449, 95% CI = 1.023–2.054, *P* = 0.037), vascular invasion (HR = 1.962, 95% CI = 1.441–2.728, *P* < 0.001), p53 positivity (HR = 1.563, 95% CI = 1.131–2.157, *P* = 0.007), and submucosal tumor infiltration (HR = 1.274, 95% CI = 1.033–1.571, *P* = 0.024) were significantly associated with patient survival time. Other variables had P values greater than 0.05, indicating that they did not have significant predictive ability in the univariate model. We then performed a multivariate Cox regression analysis considering all variables. In the multivariate model, poorly differentiated tumor (HR = 1.514, 95% CI = 1.142–2.134, *P* = 0.021), vascular invasion (HR = 1.778, 95% CI = 1.389–2.633, *P* < 0.001), p53 positivity (HR = 1.801, 95% CI = 0.907–2.243, *P* = 0.062), and submucosal tumor infiltration (HR = 1.189, 95% CI = 0.968–1.355, *P* = 0.077) had P values less than 0.05, indicating that they still had significant predictive ability in the multivariate model. Among them, vascular invasion had the largest HR (HR = 1.778), indicating that it had the most significant impact on patient survival time **(**Table 2**).**

**Table 2:** Univariate and multivariate analysis of overall survival (OS) in Gastric cancer (GC) patients underwent ESD/ gastrectomy in training cohort

	Univariate analysis			Multivariate analysis		
	P	HR	95% confidence interval	P	HR	95% confidence interval
**Gender** male/female	0.219	0.847	0.648–1.107			
**Age** > 60 y/≤60 y	0.091	1.027	0.996–1.059			
**ASA** II/I	0.316	1.172	0.861–1.593			
**ECOG PS** 1/0	0.123	1.234	0.945–1.614			
**Family history of gastric cancer** Yes/No	0.066	1.386	0.979–1.963			
**Tumor size** > 2.0 cm/≤2.0 cm	**0.024**	1.569	1.062–2.318	0.084	1.482	0.977–2.184
**Differentiation** Poorly/ high + Moderately	**0.037**	1.449	1.023–2.054	0.021	1.514	1.142–2.134
**Vascular invasion** Yes/No	**< 0.001**	1.962	1.441–2.728	< 0.001	1.778	1.389–2.633
**P53** Yes/No	**0.007**	1.563	1.131–2.157	0.062	1.801	0.907–2.243
**Ki-67** ≥ 50%/<50%	0.283	1.186	0.863–1.630			
**Tumor infiltration** Submucosa/ Mucosa	**0.024**	1.274	1.033–1.571	0.077	1.189	0.968–1.355
**MSI status** MSI-high/ (MSS/MSI-low)	0.353	0.856	0.613–1.195			
**Treatment method** ESD/ gastrectomy	0.133	1.121	0.981–1.235			

ESD: Endoscopic Submucosal Dissection; ASA:American Society of Anesthesiologists;ECOG:Eastern Cooperative Oncology Group; CTC: Circulating Tumor Cells

TNM stages are according to AJCC 8th edition

.

### Univariate and multivariate Cox regression analysis of predictors for gastric cancer patient recurrence time

In univariate analysis, tumor size (> 2.0 cm vs. ≤2.0 cm), differentiation (poorly vs. high + moderately differentiated), vascular invasion (yes vs. no), p53 status (yes vs. no), tumor infiltration (submucosa vs. mucosa), and treatment method (ESD vs. gastrectomy) were statistically analyzed. Among these factors, tumor size, differentiation, vascular invasion, p53 status, and tumor infiltration showed statistically significant associations with survival outcome (*P* < 0.05).

In multivariate analysis, only tumor size, differentiation, vascular invasion, and p53 status remained significant prognostic factors after adjusting for other factors. This suggests that these four factors are independent predictors of survival outcome in patients with gastric cancer. Specifically, patients with poorly differentiated tumors, tumors with vascular invasion, and tumors with positive p53 expression had a significantly worse prognosis compared to their counterparts with high/moderately differentiated tumors, tumors without vascular invasion, and tumors with negative p53 expression, respectively (Table 3).

**Table 3:** Univariate and multivariate analysis of recurrence-free survival (RFS) in Gastric cancer (GC) patients underwent ESD/ gastrectomy in training cohort

	Univariate analysis			Multivariate analysis		
	P	HR	95% confidence interval	P	HR	95% confidence interval
**Gender** male/female	0.176	1.129	0.940–1.357			
**Age** > 60 y/≤60 y	0.062	1.201	0.989–1.458			
**ASA** II/I	0.482	1.069	0.848–1.345			
**ECOG PS** 1/0	0.347	0.892	0.692–1.147			
**Family history of gastric cancer** Yes/No	0.079	1.256	0.971–1.623			
**Tumor size** > 2.0 cm/≤2.0 cm	**0.019**	1.395	1.063–1.832	0.091	1.241	0.927–1.663
**Differentiation** Poorly/ high + Moderately	**0.029**	1.342	1.032–1.746	0.037	1.286	1.017–1.624
**Vascular invasion** Yes/No	**< 0.001**	2.014	1.548–2.616	< 0.001	1.852	1.381–2.454
**P53** Yes/No	**0.006**	1.546	1.133–2.114	0.023	1.389	1.047–1.844
**Ki-67** ≥ 50%/<50%	0.301	1.153	0.896–1.484			
**Tumor infiltration** Submucosa/ Mucosa	**0.018**	1.405	1.065–1.856	0.052	1.224	0.999–1.501
**MSI status** MSI-high/ (MSS/MSI-low)	0.485	1.114	0.844–1.472			
**Treatment method** ESD/ gastrectomy	0.102	1.200	0.973–1.479			

ESD: Endoscopic Submucosal Dissection; ASA:American Society of Anesthesiologists;ECOG:Eastern Cooperative Oncology Group; CTC: Circulating Tumor Cells

TNM stages are according to AJCC 8th edition

### The OS and RFS of the ESD versus surgery in EGC patients

**Fig. 2:**
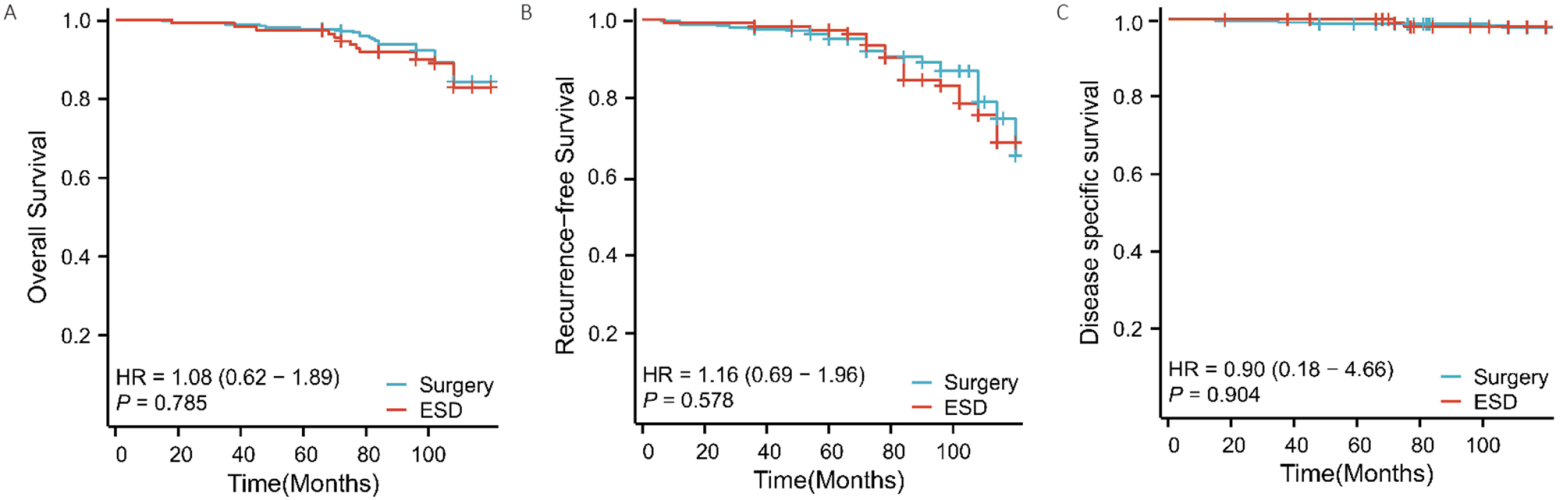
Overall survival (OS), recurrence-free survival (RFS) and disease specific survival (DSS) after surgery in the cohort of patients with early gastric cancer treated with surgery and ESD according to the different groups of treatment modalities (Fig. 2A represents overall survival; Fig. 2B represents recurrence-free survival; Fig. 2C represents disease specific survival)

The log-rank test was used to compare the prognosis of early gastric cancer between the ESD and surgery groups. For the overall survival rate, as shown in Fig. 2A, there was no statistically significant difference between the two groups (*P* = 0.785). In the ESD group, the 1-year, 3-year, and 5-year overall survival rates were 99.1%, 98.2%, and 96.4%, respectively, with a median survival time of 48.0 months. In the surgery group, the 1-year, 3-year, and 5-year overall survival rates were 100.0%, 98.8%, and 97.6%, respectively.

As shown in Fig. 2B, there was also no statistically significant difference in the recurrence-free survival rate between the two groups (*P* = 0.578). In the ESD group, the 1-year, 3-year, and 5-year recurrence-free survival rates were 98.2%, 93.5%, and 90.2%, respectively.In the surgery group, the corresponding rates were 98.5%, 97.6%, and91.0%, respectively. For the patients who experienced recurrence in the ESD group (26 in total), 11 of them underwent subsequent surgical treatment.

As shown in Fig. 2C, there was also no statistically significant difference in the Disease-free survival rate between the two groups (*P* = 0.904). In the ESD group, the 1-year, 3-year, and 5-year recurrence-free survival rates were 100.0%, 100.0%, and 99.0%, respectively.In the surgery group, the corresponding rates were 99.6%, 98.8%, and 97.6%, respectively.**(**Fig. 2**)**.

### Comparison of postoperative complications between patients in the ESD and Surgical groups

The ESD group consisted of 262 patients, with a total of 13 cases (5.0%) of postoperative complications observed. In contrast, the surgery group included 277 patients and had a higher incidence of postoperative complications, with a total of 21 cases (7.6%).

Specifically, the ESD group had lower incidence rates of gastric perforation (2 cases, 0.8%), bleeding (1 case, 0.4%), infection (1 case, 0.4%), pulmonary infection (0 cases), diarrhea (0 cases), arterial thrombosis (0 cases), venous thrombosis (0 cases), respiratory distress (0 cases), and arrhythmia (0 cases) compared to the surgery group **(**Table 4**)**.

**Table 4:** Comparison of postoperative complications between the ESD and surgery groups

Postoperative complications	ESD group (*n* = 262)	Surgery group (*n* = 277)	P-Value
Gastric perforation	2 (0.8)	17(20.9)	0.156
Bleeding	1 (0.4)	5 (1.8)	0.352
Infection	1 (0.4)	3 (1.1)	0.467
Delayed gastric emptying	0(0.0)	2 (0.7)	0.496
Pulmonary infection	0(0.0)	2 (0.7)	0.499
Diarrhea	0(0.0)	0(0.0)	1.000
Arterial thrombosis	0(0.0)	1(0.4)	0.496
Venous thrombosis	0(0.0)	0(0.0)	1.000
Respiratory distress	0(0.0)	1(0.4%)	0.496
Arrhythmia	0(0.0)	0(0.0)	1.000
Total	4 (1.5%)	21 (7.6%)	0.012

## Discussion

In this study, we compared the clinical outcomes of endoscopic submucosal dissection (ESD) versus surgical resection in patients with early gastric cancer. Our results showed that there were no significant differences between the two groups in terms of overall survival (OS) or recurrence-free survival (RFS), also patients in the ESD group had fewer postoperative complications. These findings indicate that ESD may be a safe and effective alternative to surgery for the treatment of early gastric cancer.

ESD has become a major treatment modality for specific early gastric cancer (EGC) patients who meet the absolute and expanded criteria in China [3, 9–11]. Similarly, in other Asian centers, a multicenter study by Ryu et al [12]. comparing the long-term efficacy of endoscopic submucosal dissection (ESD) and surgical resection for early gastric cancer showed that ESD may be an acceptable and effective treatment option compared to surgical resection, due to its lower incidence of early complications and shorter hospital stays. However, the authors note that patients undergoing ESD have a higher incidence of metachronous lesions, which may be both an advantage and a disadvantage of preserving the gastric mucosa. While ESD may be less costly for treating EGC patients, strict endoscopic surveillance for more than 5 years is strongly recommended for those treated with ESD, as the cost of treatment may be higher if recurrence occurs. At the same time, there are a series of unique surgical complications for patients after gastric cancer surgery, such as postoperative intussusception [13]. For early gastric cancer patients, those who undergo endoscopic submucosal dissection (ESD) or surgery exhibit longer survival rates. Therefore, the causes of death during subsequent follow-up periods are worth noting. We identified two primary causes of death. Due to the extended follow-up duration, a considerable number of patients succumbed to age-related diseases that emerged with increasing age. Additionally, some patients experienced local recurrence post-surgery, which was also a contributing factor to eventual mortality, consistent with previous research [14, 15]. Hence, we plotted the disease-specific survival (DSS) curve. Interestingly, we observed that specific deaths due to tumor recurrence were relatively rare. Consequently, both ESD and surgical interventions offer favorable prognoses for patients.

Our univariate and multivariate Cox regression analyses identified several factors associated with patient survival and recurrence time, including tumor size, differentiation, vascular invasion, p53 positivity, and submucosal tumor infiltration. In the multivariate model, poorly differentiated tumor, vascular invasion, p53 positivity, and submucosal tumor infiltration remained significant predictors of patient survival time, with vascular invasion having the largest hazard ratio. For recurrence time, only tumor size, differentiation, vascular invasion, and p53 status were significant prognostic factors. Zou et al [16]. concluded that CTC is also a risk factor for patients with postoperative gastric cancer and can be performed in patients with ESD or surgically treated early gastric cancer if necessary to increase the prediction of postoperative recurrence as well as survival.

Our study has some limitations. First, it was a retrospective analysis of data from a single institution, which may limit the generalizability of our findings. Second, we did not consider other important factors such as socioeconomic status, which could have influenced survival outcomes. Third, although there were no statistically significant differences between the baseline data of the ESD group and the surgery group as indicated in the baseline characteristics table, there are some variables, such as surgical difficulty, that lack explicit metrics, potentially leading to selection bias. At the same time, due to differences in treatment selection, SM2 and SM3 cases were excluded (although they were few in number).

In conclusion, our study provides evidence supporting the consideration of ESD as a potential treatment option for early gastric cancer. While our findings suggest comparable prognostic outcomes between surgery and ESD, it is essential to emphasize that treatment modalities should be chosen based on individual patient characteristics and tumor profiles, considering established indications and guidelines. The importance of careful patient selection in predicting survival and recurrence outcomes is underscored by our results. Further research is necessary to validate our findings and explore additional factors influencing the choice between surgery and ESD for the treatment of early gastric cancer.

## Data Availability

The datasets used and analyzed during the current study are available from the corresponding author on reasonable request.

## Abbreviations

GC: gastric cancer
ESD: Endoscopic Submucosal Dissection
ASA: American Society of Anesthesiologists
CTC: Circulating Tumor Cells
ECOG: Eastern Cooperative Oncology Group
PS: performance status
MSI: microsatellite instability
MSS: microsatellite stable
CT: Computed Tomography
MRI: Magnetic Resonance Imaging
HR: Hazard ratio
OS: overall survival
RFS: recurrence-free survival
EMR: endoscopic mucosal resection
